# Supplementary material for: Complexome profiling of the Chlamydomonas psb28 mutant reveals TEF5 as an early PSII assembly factor
Source: Plant Cell. 2025 Mar 17;37(6):koaf055. doi: 10.1093/plcell/koaf055 (PMC12164590; doi:10.1093/plcell/koaf055)
Supplement: koaf055_Supplementary_Data [file koaf055_supplementary_data.zip › psb28_interactive.zip!/plots/intensity/qualityPlots/peptides/CGL139_Cre16.g679300.t1.2.html]

### Cre16.g679300.t1.2

Trivial name: CGL139  
SeqCoverage [median]: 26.20  
Mass: 15.49 kDa  
  
 

| Sequence | MissedCleavage | Mass [Da] | wtIntensity | muIntensity | Score |
| --- | --- | --- | --- | --- | --- |
| GVNGFNEEAER | 0 | 1220.54 | 0.00 | 2183.81 | 90.83 |
| GVNGFNEEAERQDKLDGYL | 2 | 2153.00 | 0.00 | 3254.09 | 41.35 |
| TPPTVPGSEDELPPWVRR | 1 | 2032.04 | 3285.63 | 6671.51 | 75.57 |
|  |  |  | 3285.6 | 12109.4 |  |
